# A Dataset for Depth in Robotic Endoscopy with Dynamic Scenarios (DRENDS)

**DOI:** 10.1038/s41597-026-07775-2

**Published:** 2026-08-10

**Authors:** Gerardo Loza-Galindo, Mattia Magro, Benjamin Calmé, Junlei Hu, Emanuele Ruffaldi, Dominic Jones, Elena De Momi, Sharib Ali, Pietro Valdastri

**Affiliations:** 1 School of Computer Science, University of Leeds, Leeds, LS2 9JT UK; 2 Politecnico di Milano, Piazza Leonardo da Vinci, Milano, 32, 20133 Italy; 3 Medical Microinstruments, Inc., 5022 Gate Parkway, Jacksonville, 32256 FL USA; 4 School of Electronic and Electrical Engineering, University of Leeds, Leeds, LS2 9JT UK

## Abstract

Depth perception in robotic minimally invasive surgery remains a critical challenge for many downstream tasks, demanding advanced depth estimation techniques and ground truth data for their validation. Current datasets lack data with ground-truth depth information in dynamic scenarios; therefore, we present DRENDS (Depth in Robotic Endoscopy with Dynamic Scenarios)^1^, a novel dataset comprising sequences of high-resolution stereo images captured during the robotic laparoscopic manipulation of a human phantom and *ex vivo* porcine tissue, along with ground-truth point clouds for each frame and calibration data. The data were collected under three illumination conditions and across different anatomies involving tissue manipulation and non-rigid deformations. Our code for rectifying stereo images, handling camera-perspective occlusions, and obtaining depth maps per frame is open source for reproducibility and easy adaptation. Finally, we also conduct baseline evaluations using state-of-the-art depth estimation models to establish benchmark performance on our dataset. The results and data highlight the challenges and potential of metric temporally consistent depth estimation in robotic surgery, encouraging further advancements in tissue deformation prediction for medical applications. We publicly release DRENDS^1^ to foster innovation and collaboration in this critical field.

## Background & Summary

Surgical scene understanding is driven by the analysis of surgical data, with depth estimation being one of the key pillars for developing solutions in many downstream tasks that can further assist the clinical team in the surgical room^2^. In robotic minimally invasive surgery (RMIS), analysing laparoscopic images to obtain reliable depth estimation can enable force estimation, 3D reconstruction, augmented reality, and autonomy^3,4^. The integration of active sensors that facilitate 3D information, such as laser-based systems (e.g., Light Detection and Ranging (LiDAR) and Time-of-Flight (ToF) sensors), is often costly and complex due to the nature of the procedures. The workspace in RMIS procedures is limited by the volume of the inflated abdominal area, which restricts manoeuvrability, increases the risk of tissue interference, and demands small, low-range sensors^5,6^. Consequently, the use of passive sensors (RGB cameras) has provided a promising solution that relies heavily on the availability of data with 3D ground truth for algorithm validation and, when using deep learning techniques, for the training of intelligent algorithms^7^. Approaches in this domain must be robust to the challenges in the surgical environment, such as blurry images, varying illumination, tissue deformations, and homogeneous texture. Therefore, acquiring laparoscopic images with reliable depth information is crucial for developing new technologies.

Several publicly available datasets have supported research in surgical depth estimation, each offering unique contributions while presenting certain limitations. **SERV-CT**^8^ comprises 16 stereo-endoscopic image pairs from *ex vivo* porcine samples, each accompanied by anatomical segmentations derived from cone-beam computed tomography (CT) scans. This dataset provides a reliable benchmark due to its high-quality ground truth and detailed anatomical references, albeit with limited diversity and size. The **Hamlyn Laparoscopic/Endoscopic Dataset**^9^ provides laparoscopic and endoscopic video sequences, with a subset containing ground-truth depth maps generated using the Libelas stereo matching software on *in vivo* data. As one of the earliest resources in this field, it remains widely used for benchmarking despite its limited accuracy in depth annotation. **EndoAbs**^10^ consists of stereo images and point clouds acquired through a 3D scanner of 3D-printed abdominal organ phantoms. It offers 120 non-sequential, low-resolution frames (640×480), under challenging conditions such as variable lighting and simulated smoke. This makes it valuable for assessing algorithm robustness, although its size and resolution restrict broader applicability. **SCARED**^11^ dataset provides nine samples, each comprising 4 to 5 short stereo frame sequences. The first frame of each sequence provides high-precision depth information through a structured light approach. On subsequent frames, recorded camera movements and fixed scenarios are leveraged to transform 3D information into their corresponding camera views. This design enables multi-view consistency studies. **EndoSlam**^12^ provides monocular colonoscopic video sequences accompanied by 6-degree-of-freedom camera poses and dense 3D map ground truth from 3D scanned porcine cadaver organs. The dataset supports the development of visual SLAM techniques in static challenging surgical environments, with a primary focus on colonoscopy. **SimCol3D**^13^ is a synthetic colonoscopy dataset generated from multiple trajectories within static 3D colon models. It provides perfectly aligned depth maps and camera poses, offering a controlled environment for algorithm testing. **C3VD**^14^ introduces stereo video data of realistic high-fidelity phantom models alongside 3D information per-frame acquired from a corresponding virtual environment. The dataset contains sequences along diverse sections of the colon. By combining realistic surgical imaging with precise geometric annotations, C3VD^14^ provides a valuable benchmark for both frame-wise and sequence-based approaches to depth estimation. Table 1 presents a surmised comparison of all datasets, including our contribution, which is detailed in the rest of this work.

**Table 1:** Comparison of publicly available datasets for depth estimation in endoscopy or minimally invasive surgery.

Dataset	Modality	Data type	Data size	Image resolution	Frame rate	Ground Truth	Camera Motion	Tissue Motion
Hamlyn^9^	Stereo Laparoscopy	*In vivo*	40,000 images	384×192	unspecified	Libelas stereo matching	True	True
EndoAbs^10^	Stereo Laparoscopy	Phantom	120 images	640×480	3.3 fps	3D scanner	False	False
SCARED^11^	Stereo Laparoscopy	*Ex vivo* (porcine)	22,950 images	1280×1024	20 fps	Structure from light	True	False
SERV-CT^8^	Stereo Laparoscopy	*Ex vivo* (porcine)	16 images	720×576	none	CT Scan	True	False
EndoSLAM^12^	Mono colonoscopy	*Ex vivo* (porcine)	42,700 images	1350×1080 1280×720 640×480 320×320 256×256	3–35 fps	3D scanners	True	False
SimCol3D^13^	Mono colonoscopy	Simulation	37,800 images	475×475	20 fps	Virtual Env.	True	False
C3VD^14^	Stereo colonoscopy	Phantom	30,073 images	1350×1080	25 fps	Virtual Env.	True	False
DRENDS^1^ (ours)	Stereo Laparoscopy	Phantom & *ex vivo* (porcine)	34,650 images	1920×1080 1280×720	16 fps	Fast ToF camera	False	True

Additionally, datasets developed for other surgical vision tasks also highlight the importance of capturing realistic tissue deformation. For example, StereoMIS^15^ provides stereo endoscopic images with camera pose estimates derived from robot kinematics during *in vivo* procedures involving breathing motion and tissue manipulation or resection. Similarly, STIR^16^ introduces a dataset for surgical point tracking in deformable scenes using infrared-visible imagery. While these datasets represent valuable contributions and highlight the importance of capturing realistic surgical dynamics, the lack of ground-truth depth or 3D geometry limits their suitability for the quantitative evaluation of reconstruction methods.

The current state of the art in laparoscopic depth estimation has benefited significantly from datasets in Table 1, most of which provide RGB images and ground-truth depth maps along with camera poses for different camera views. They aim to provide a reliable framework for assessing the performance of algorithms that estimate depth maps per frame and algorithms that stitch those maps to reconstruct the surgical scenario. However, existing datasets simplify the challenge of depth acquisition in surgical environments by fixing tissue locations and assuming rigid properties. In practice, tissue motion and non-rigid deformations often arise from surgical manipulation and physiological activity. To date, no dataset has captured depth information under these dynamic conditions. Such data are crucial for improving depth perception approaches and validating their applicability in real surgical settings.

To address these limitations, we introduce DRENDS^1^, a dataset of stereo image sequences captured with a Da Vinci surgical system and paired with ground-truth point clouds from a synchronised real-time time-of-flight camera. The resource includes video sequences (RGB, point clouds and intensity images) recorded at 16 frames per second (FPS) under three illumination conditions across different anatomies undergoing manipulation and non-rigid deformation. On top of that, timestamps for each frame, calibration data, and code to generate depth maps for both left- and right-camera views are released to ensure reproducibility and facilitate adaptation by the research community. Table 1 also includes our dataset, highlighting its contribution of high-resolution RGB images and depth maps acquired under tissue motion at clinically relevant frame rates. The method and result for the camera calibration are also reported. For further validation, we benchmark state-of-the-art (SOTA) depth estimation models using standard spatial accuracy metrics, as well as a temporal metric enabled by the sequential nature of the data. These results establish a reference point for future studies and highlight the unique challenges of depth estimation in the presence of tissue deformations during robotic-assisted laparoscopic surgery.

## Methods

We introduce a novel dataset for Depth in Robotic Endoscopy with Dynamic Scenarios (DRENDS dataset)^1^, specifically designed for scenarios involving realistic tissue deformations. DRENDS^1^ dataset comprises synchronised stereo videos and corresponding ground-truth (GT) depth information that capture tissue deformations during manipulation. To our knowledge, this is the first dataset that includes high-resolution videos of dynamic surgical scenes (i.e., complex tissue deformations) with ground truth depths. The methodology for generating the dataset is organised into four parts: setup design, calibration, acquisition protocol, and data processing.

### Setup

**Fig. 1:**
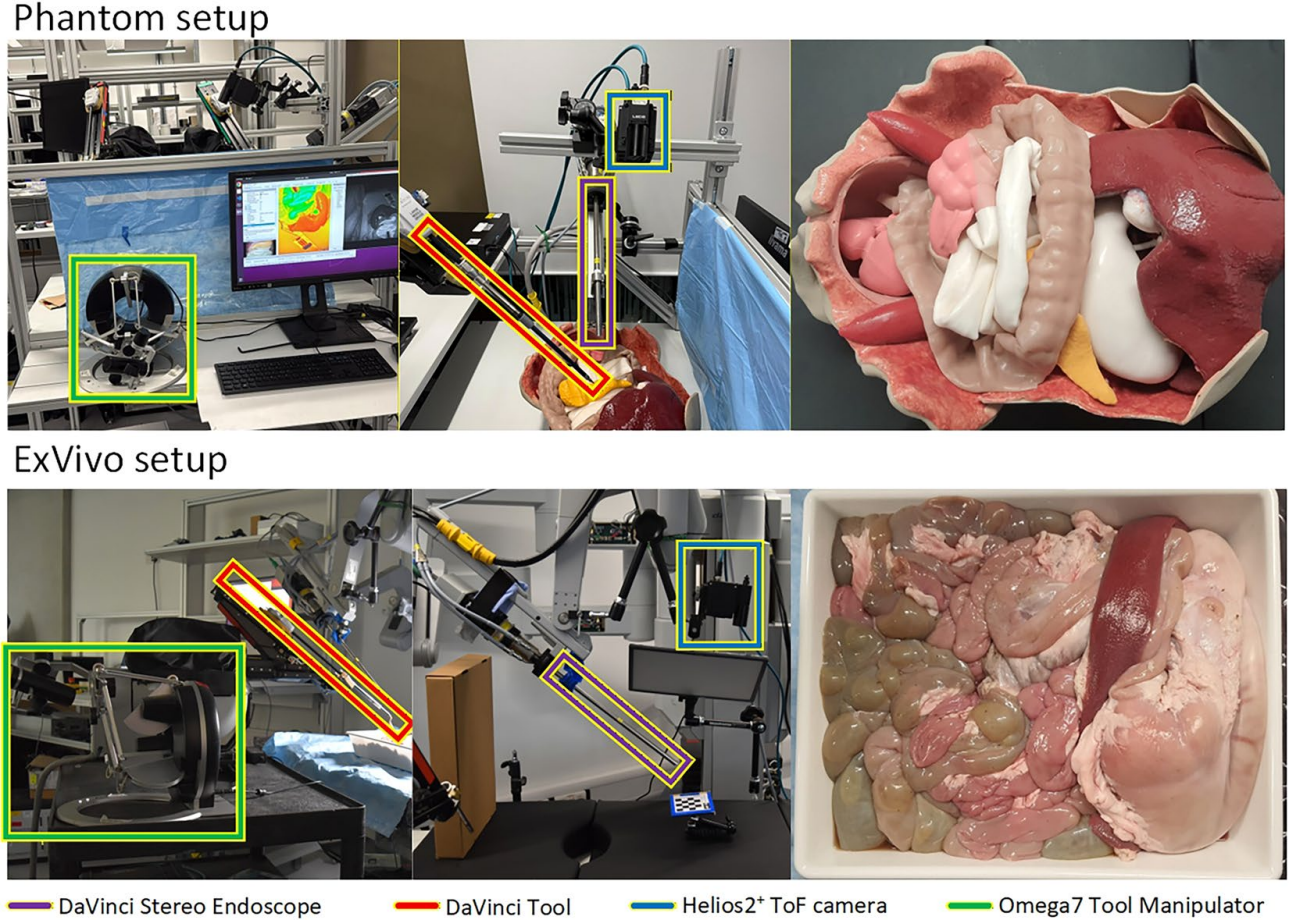
Phantom and *ex vivo* setup, first and second row, respectively. The operator controls the DaVinci tool using the Omega7 device as a tool manipulator; the endoscopic and helios camera views are displayed on a screen next to him and are used for visual feedback during manipulation.

The dataset was acquired at the University of Leeds using the Da Vinci Research Kit (dVRK) with the Da Vinci Si, Intuitive Surgical, Sunnyvale, CA, USA. The hardware setup consisted of: (i) one Da Vinci robotic arm, (ii) an Omega7 haptic device (from Force Dimension, inc. Nyon, Switzerland) as tool manipulator, (iii) the stereo endoscopic camera from the Da Vinci system (Ikegami 720p cameras in the phantom setup and 1080i Panasonic cameras in the *ex vivo* setup), and (iv) a Helios2^+^ ToF camera from LUCID Vision Labs Inc. Burnaby, Canada. Figure 1 illustrates the configurations for the phantom and *ex vivo* data collections. The stereo and ToF cameras were mounted to maximise the coverage workspace, with the stereo camera positioned so that it did not obstruct the Helios2^+^ camera view. All devices were interfaced through the Robot Operating System (ROS). During data acquisition, an operator manipulated the tissues with the robotic tool via the tool manipulator device.

### Calibration

The intrinsic parameters of the Helios2^+^ ToF camera were obtained using the Arena Software Development Kit provided by LUCID Vision Labs. The intrinsic parameters of the stereo endoscopic cameras were estimated with images of a planar chessboard calibration pattern (Grid size: 3 × 4 squares, Square size: 15 × 15 mm.), using the OpenCV library with the Python interface. Leveraging Zhang^17^’s method for camera calibration first, individual monocular calibrations for the left and right cameras were performed, optimising the intrinsic matrices and distortion coefficients using a nonlinear least-squares algorithm with a termination criterion of 500 iterations or $\epsilon {=10}^{-8}$. Initial guesses for the focal length and principal point were set from the image dimensions, and intrinsic parameters were refined while distortion terms remained unconstrained. In the second stage, stereo calibration was carried out using the previously estimated intrinsics as fixed initial values and refining the rotation (${{\bf{R}}}_{stereo}$) and translation (${{\bf{T}}}_{stereo}$) between the cameras. Stereo rectification was applied to all images using the computed projection matrices.

To align the two sensing modalities, the calibration pattern was also captured with the ToF camera, and we estimated the rigid transformation between their coordinate systems using a nonlinear least-squares optimisation in Python. Given 3D points in the depth-camera frame, ${{\bf{X}}}_{i}^{{\rm{PC}}}\in {{\mathbb{R}}}^{3}$, and corresponding RGB image points $${{\bf{u}}}_{i}=[\begin{array}{c}{u}_{i},{v}_{i}\end{array}]$$, we estimate the rotation ${\bf{R}}\in SO(3)$ and translation ${\bf{T}}\in {{\mathbb{R}}}^{3}$ that map depth-camera points into the RGB camera frame. The transformation of the points is given by ${\hat{{\bf{X}}}}_{i}^{{\rm{RGB}}}={\bf{R}}{{\bf{X}}}_{i}^{{\rm{PC}}}+{\bf{t}}$, and the projection onto the image plane is ${\hat{{\bf{u}}}}_{i}=\pi ({\bf{K}},{\bf{R}}{{\bf{X}}}_{i}^{{\rm{PC}}}+{\bf{t}})$ where *π*(·) denotes the pinhole projection. Finally, the calibration solves the optimisation described by the Eq. 1. Calibration accuracy was assessed through both the mean 2D reprojection error (in pixels) and the 3D alignment error (in millimetres), with detailed results reported in the Technical Validation section.

1 $$({{\bf{R}}}^{\ast },{{\bf{t}}}^{\ast })=\mathrm{arg}\,\mathop{\min }\limits_{{\bf{R}},{\bf{t}}}\mathop{\sum }\limits_{i=1}^{N}{\Vert {{\bf{u}}}_{i}-\pi ({\bf{K}},{\bf{R}}{{\bf{X}}}_{i}^{{\rm{PC}}}+{\bf{t}})\Vert }_{2}^{2}.$$

### Acquisition protocol

Each organ — colon, intestine, stomach, liver, and pancreas — was recorded under three illumination conditions (high setting the Da Vinci light source to the 80% of its power, standard setting the Da Vinci light source to the 60% of its power, and external using a white light source parallel to the camera view of the endoscope). High illumination camera created specular highlights that reduced visible texture, standard illumination represented optimal viewing conditions, and external illumination was used to recreate scenarios where illumination is limited, yet the scene remains visible.

Two acquisition strategies were used: sequence and video. In the sequence approach, 50 non-consecutive frames are captured, each corresponding to a distinct tissue deformation, allowing a broader range of deformation states to be recorded. In the video approach, frames are recorded continuously at 16 FPS while tissues are manipulated to transition between different deformation states. The two acquisition strategies are performed independently, and the sequences are not subsampled from the videos.

#### Phantom setup

Data were collected using the synthetic abdominal organs of the EasySurg Training System from XCorpa medical simulators. Together, 15 sequences and 15 videos of 90 seconds total 23,250 frames. The stereo and depth cameras were synchronised using the timestamps of the computer subscribed to all the ROS nodes and the ROS-Python time synchroniser function.

#### *Ex vivo* setup

Data were collected using porcine *ex vivo* tissues (without pancreas). Together, 12 sequences and 12 60-second videos add up to 12,120 frames. To handle the interlace of the 1080i camera, a simple deinterlacing function was built for the endoscopic image publisher, dropping one channel and interpolating from the vertical neighbours’ pixels. The acquisition software was built in ROS. This setup relies on the presynchronisation of the clocks in the capture computers, so a post-collection routine was run to filter videos at 16 FPS and ensure synchronised frames from all cameras.

The following protocol was followed in each acquisition (phantom sequence, phantom video, *ex vivo* sequence and *ex vivo* video):

1. Position the synthetic organ within the phantom framework.
2. Adjust the phantom so that the target organ is centred in the endoscopic view.
3. Bring the surgical tool into the field of view.
4. Set the illumination condition.
5. Test the robotic system connectivity. The trained operator verifies that the tool moves correctly within the camera’s view.
6. Start recording.
7. Tissue manipulation:
  - During video collection, the operator explores the tissue surface, going from one tissue deformation to another and maximising the visible surface.
  - During sequence collection, the operator positions the tissues under different deformations relative to the previous frame and stays still until a second operator manually triggers the capture of all cameras.
8. Stop recording.
9. If there are consecutive frames in a video with a time-step difference greater than 0.1875 seconds during a tissue manipulation, the video was discarded and re-taken.

### Data processing

To generate the ground-truth depth maps, we applied a multi-stage processing pipeline to the raw point clouds from the ToF camera. The main steps included noise filtering to handle inconsistencies and invalid pixels, masking occlusions, and refining coordinate transformation to align the point cloud with the stereo camera view.

#### Noise filtering

**Fig. 2:**
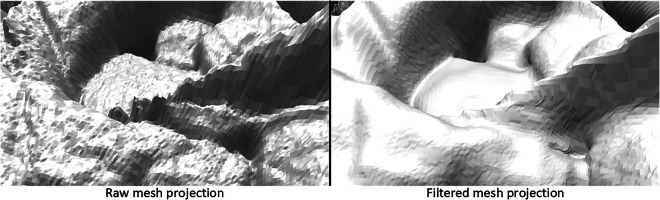
Comparison of the reconstructed mesh before and after the noise filter.

We start by removing the null and infinite values from the raw ToF point clouds, which were replaced by interpolation from the nearest neighbours. Then a temporal smoothing was applied to depth values, using a sliding window of ±1 sec. (≈16 frames). Pixel values were smoothed only iwhen depth differences fell within the tolerance error reported by the Helios2^+^ manufacturer (±4 mm), thereby preserving true depth changes due to tissue motion. Finally, a spatial filter was used; each frame was smoothed using a bilateral filter^18^ (OpenCV implementation) on a sliding window of 12 × 12 pixels, which preserves depth discontinuities while reducing local noise. The parameters used for temporal and spatial filtering were empirically selected based on qualitative evaluation of representative sequences, following common practice in depth-map denoising to balance noise reduction with preservation of depth discontinuities and deformation-induced geometric changes. Filtered point clouds were used to generate surface meshes of the scene. Figure 2 shows an example of the mesh with and without the filters applied to the point cloud.

#### Occlusion mask generation

**Fig. 3:**
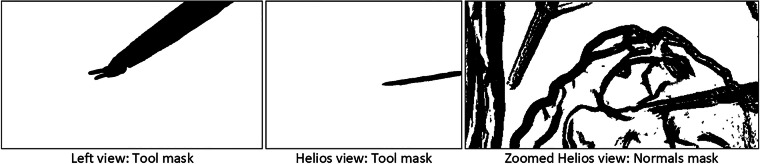
Mask to filter out valid surfaces on the meshes that generate depth ground truth. Tool masks in the first and second columns. Normals mask in the third column, it leaves out points that do not capture the location of the surface on the laparoscopic camera view because they were occluded on the Helios camera view.

Invalid pixels due to occlusion were identified based on two criteria. First, surgical tools were masked out because their reflective, thin surfaces led to unreliable depth values. Tools were segmented in each camera view using the Segment Anything Model v2^19^ (SAM2), with manually selected image points as prompts (Fig. 3 first and second columns). The prompts used in our pipeline are included in the resources shared with our dataset to ensure reproducibility. Second, to account for viewpoint differences between the Helios2^+^ and the endoscopic cameras, we removed surface elements that are not visible from the endoscopic perspective. To achieve this, surface normals were computed for each vertex of the Helios mesh before transformation. Vertices whose normals were perpendicular to the Helios z-axis were identified as belonging to surfaces that fall outside the endoscopic field of view and were therefore considered occluded. These regions were subsequently removed from the final depth maps (Fig. 3, third column).

#### Coordinate transformation

**Fig. 4:**
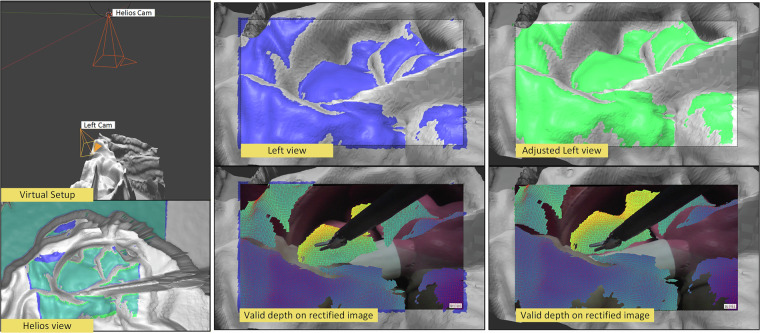
Transformation pipeline. The virtual setup set the cameras’ positions according to the calibration results (first column). The left view shows the results of re-projecting the mesh onto the left image plane (second column). After manually adjusting the left camera position within the error margin, the transformation to move the camera is stored and used to produce new re-projections (third column). Notice that the left camera views of the virtual environment show the pixels with valid depth information before and after the adjustment, blue and green, respectively.

The transformation from Eq. 1 on the meshes from the ToF camera to the stereo camera-coordinated systems demonstrates some misalignment when depth is rendered and overlaid on the RGB information (second column in Fig. 4). Therefore, to account for residual misalignment within the transformation error margin (error defined by the mean difference between the triangulated and transformed 3D points $e=\frac{1}{N}\sum ||{{\bf{X}}}_{i}^{{\rm{RGB}}}-{\hat{{\bf{X}}}}_{i}^{{\rm{RGB}}}{||}^{2}$), we defined a compensation transformation. This was obtained by replicating the setup in a 3D modelling environment (Blender) and manually adjusting the endoscopic camera position until the rendered and real RGB views were aligned (third column in Fig. 4). The resulting offset defines the compensation transformation, which is shared within the dataset.

#### *Ex vivo* image denoise

**Fig. 5:**
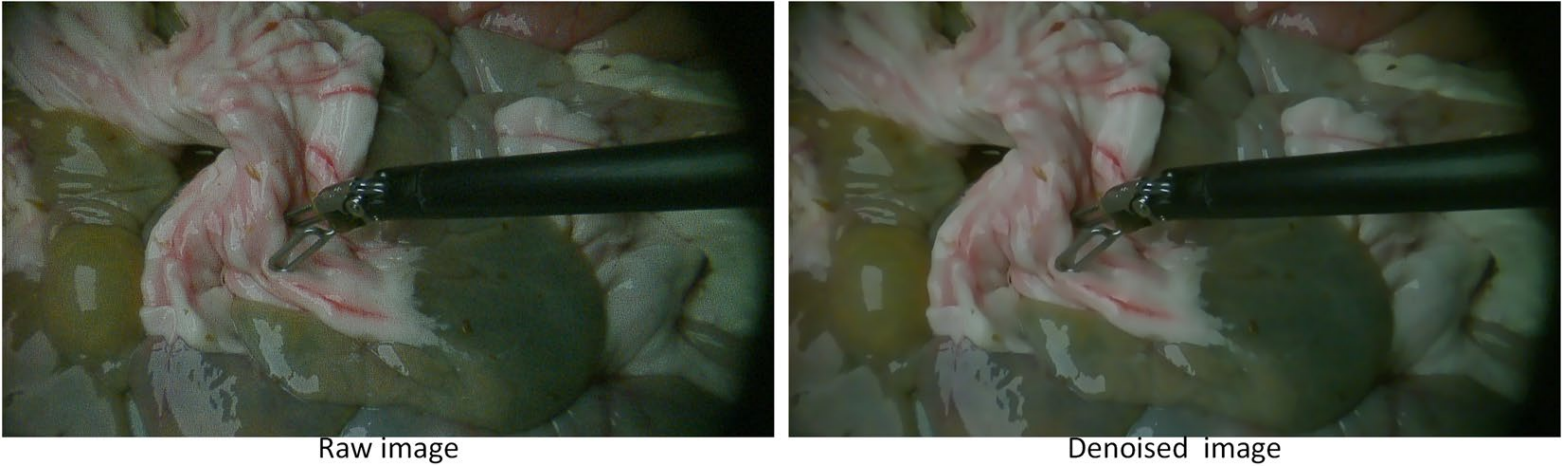
Denoising *Ex vivo* image.

The endoscopic images obtained during *ex vivo* data collection exhibit fine-grained, spatially uniform speckle noise. To denoise the images, we share the code to apply patch-based non-local means filtering^20^ with a search window of 21 × 21 pixels and a luminance strength weight of 8 to reduce luminance and chrominance noise while preserving fine tissue structure. Figure 5 shows an example of an image before and after the denoiser. Our dataset contains raw, noisy images, and the code in our repository includes the weighting, modification, and application of our denoising function.

## Data Records

**Fig. 6:**
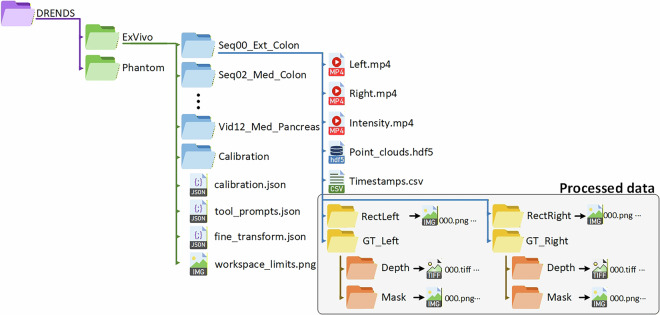
Data folder structure in DRENDS.

**Fig. 7:**
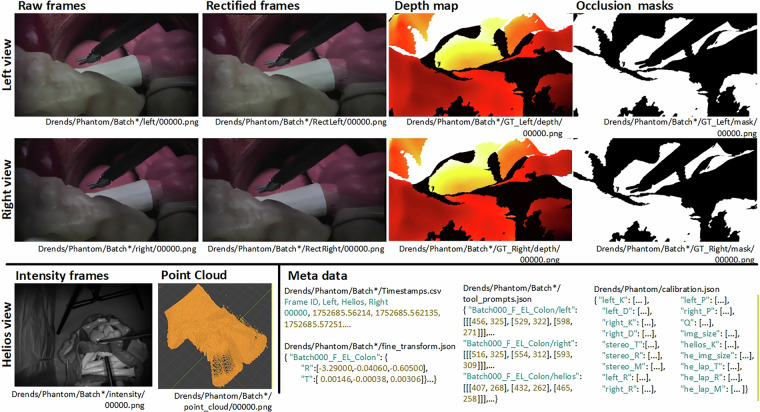
Example of the data related to a single time step in a sequence.

The DRENDS^1^ dataset is publicly available on Zenodo under the project identifier Zenodo-17598453. The data are released under a CC BY-4.0 licence to permit reuse and adaptation with attribution. The DRENDS dataset contains synchronised stereo endoscopic images, point clouds, and metadata, together with the code to generate binary masks and depth maps. Figure 6 illustrates the folder hierarchy and file formats. Data folders are named according to organ, illumination condition, and acquisition mode (sequence or video). Each recording is stored in a dedicated folder that includes the raw data: videos, point clouds, and timestamps. Once the processing pipeline is run, rectified images, masks, and depth maps are produced. File formats are consistent across the dataset: videos are .mp4, images are .png, depth maps are .tiff, point clouds are .hdf5, and timestamps are .csv. All data are stored in 16bit format. The dataset comprises 54 folders, corresponding to two modalities (phantom and *ex vivo*), five abdominal organs (colon, intestine, stomach, liver, and pancreas - liver is excluded in the *ex vivo* data), three illumination conditions (high, standard, and external) and two acquisition approaches (sequences and videos). In total, the dataset contains 34,650 frames with depth ground truth. Figure 7 shows a representative example of the data. It displays the stereo images, associated metadata and the corresponding processed depth maps and masks. This example illustrates the type and quality of the information available for each time step in the sequences.

## Technical Validation

### Calibration

A full stereo calibration was performed for each setup to ensure accurate geometric reconstruction between the left and right cameras using checkerboard images. The resulting parameters were used to triangulate the pattern’s points. The 3D information from the ToF camera is then aligned to the triangulated pattern’s points. Table 2 shows the results of the calibration and the 3D alignments. The calibration results are used to initialise the virtual environment and obtain a fine transformation that aligns the 3D information with the stereo camera view.

**Table 2:** Camera calibration results.

Type	Image resolution	Camera calibration			ToF-Stereo transformation	
		*left*	*right*	*stereo*	*3D alignment error*	*2D projection error*
Phantom	1280×720 px	0.424 px	0.415 px	0.504 px	1.43 ± 0.93 mm	6.28 ± 4.00 px
*Ex vivo*	1920×1080 px	0.332 px	0.353 px	0.523 px	4.25 ± 2.21 mm	8.73 ± 5.73 px

The temporal synchronisation accuracy was quantified by measuring inter-frame timestamp differences between sensing modalities. Across the phantom videos, the average time offset between stereo pairs was 8 ms (maximum 50 ms), indicating tight synchronisation between the stereo cameras. The results on the RGB-ToF alignment reveal a capture delay in one of the videos. The video ‘Vid11_Liver_High’ contain 16 frames with a time offset ranging from 280 ms to 1,792 ms. Discarding this outlier the average time offset is 33 ms, max time offset is 131 ms. Considering the target time offset of 62 ms for an acquisition speed of 16 FPS, only 2.2 timesteps per video have a time offset higher than the target time.

Across the *ex vivo* videos, the average time offset between stereo pairs was 9 ms (maximum 72 ms), with only a single instance exceeding the nominal frame interval. The RGB-ToF alignment shows consistency, with an average offset of 12 ms and a maximum of 92 ms. On average, 2.2 time steps per video have a time offset between sensors exceeding 62 ms. Overall, the temporal offsets remain small relative to the acquisition rate for the vast majority of frames, ensuring reliable temporal alignment for dynamic scene analysis.

### Benchmark of state-of-the-art methods

To evaluate the performance of several (SOTA) depth estimation methods, we selected well-established models and applied them to the image sequences in our dataset. All models were used in their publicly released form, without additional fine-tuning or dataset-specific adaptation, to provide a fair “out-of-the-box” comparison. The evaluation focuses on both spatial accuracy and temporal consistency, using metrics described in the following subsection.

#### Evaluation metrics

To quantitatively assess the performance of depth estimation models on DRENDS, we employ a set of metrics that evaluate both spatial accuracy and temporal consistency. The metrics are computed at the pixel level over valid regions (excluding occluded pixels using the masks) and reported as averages.

**Spatial accuracy metrics**. For each frame, we denote the ground-truth depth as ${d}_{i}$ and the predicted depth as ${\hat{d}}_{i}$, where *i* indexes the valid pixels. Following common practice in depth estimation benchmarks, we report the following metrics:

- **Mean Absolute Error (MAE)** in millimetres (*mm*):
  2 $${\rm{MAE}}=\frac{1}{N}\sum _{i}|{d}_{i}-{\hat{d}}_{i}|$$
- **Root Mean Squared Error (RMSE)** in millimetres (*mm*):
  3 $${\rm{RMSE}}=\sqrt{\frac{1}{N}\sum _{i}{({d}_{i}-{\hat{d}}_{i})}^{2}}$$
- **Log RMSE**:
  4 $${{\rm{RMSE}}}_{log}=\sqrt{\frac{1}{N}\sum _{i}{(log{d}_{i}-log{\hat{d}}_{i})}^{2}}$$
- **Absolute Relative Error (AbsRel)**:
  5 $${\rm{AbsRel}}=\frac{1}{N}\sum _{i}\frac{|{d}_{i}-{\hat{d}}_{i}|}{{d}_{i}}$$
- **Threshold accuracy**: proportion of pixels for which the difference between predicted and ground-truth depth lies within a tolerance δ in mm.:

6 $$\begin{array}{ll}{\delta }_{Threshold}=\frac{1}{N}\sum _{i}\left(\max \left(\frac{{d}_{i}}{{\hat{d}}_{i}},\frac{{\hat{d}}_{i}}{{d}_{i}}\right) < {\delta }^{k}\right), & \delta =1.25,\,k\in \{0.25,0.50,0.75\}.\end{array}$$

These metrics jointly measure absolute and relative discrepancies between predicted and true depths. While MAE and RMSE quantify absolute deviations in millimetres (*mm*), ${{\rm{RMSE}}}_{log}$ and AbsRel account for proportional and scale-dependent errors. The *δ*-accuracy terms indicate the fraction of pixels within different error tolerances.

**Aligned-depth evaluation**. Because depth estimation models may produce outputs that are accurate in structure but biased in global scale or offset, we additionally report the above metrics after rescaling predictions to the ground truth. The aligned depth ${\hat{d}}_{i}^{{\prime} }$ is obtained by minimising the least-squares difference over all valid (non-occluded) pixels per sequence:

7 $$\begin{array}{ll}\mathop{min}\limits_{s,t}\sum _{i}{(s{\hat{d}}_{i}+t-{d}_{i})}^{2}, & {\hat{d}}_{i}^{{\prime} }=s\,{\hat{d}}_{i}+t,\end{array}$$

where *s* and *t* denote the scale and translation parameters, respectively. The parameters are estimated once per sequence and applied consistently to all frames within that sequence, rather than being computed independently for each frame. This choice avoids compensating frame-to-frame prediction fluctuations through independent rescaling and therefore preserves temporal consistency during evaluation. This evaluation highlights the structural fidelity of the predictions independently of global scaling errors.

**Temporal consistency metric**. While per-frame metrics such as MAE and RMSE quantify absolute depth accuracy, they do not capture the temporal stability of predictions across frames. For example, two models may achieve similar per-frame errors, yet one may produce temporally stable depth estimates while the other exhibits frame-to-frame fluctuations (flickering) due to noise or inconsistent scale estimation. In such cases, the latter would exhibit poorer temporal consistency despite comparable per-frame accuracy. Recent work in surgical vision has further shown that enforcing temporal consistency can significantly improve depth prediction performance in endoscopic settings^21^. Therefore, we propose a metric that explicitly captures this behaviour by evaluating the agreement between predicted and ground-truth depth changes over time, which can be used for both evaluation and training of depth estimation methods. Let ${d}_{i}^{(t)}$ and ${\hat{d}}_{i}^{(t)}$ denote the ground-truth and predicted depths for frame *t*. We define:

- **Depth Temporal Consistency Error (DTCE)**: This metric assesses how accurately the predicted depth differences between frames align with the true depth changes, i.e., the average per-pixel jitter of the prediction, in *mm*. Lower values indicate better temporal consistency, as they correspond to the average absolute difference between the predicted and ground-truth depth variations across consecutive frames.

8 $${\rm{DTCE}}=\frac{1}{N}\sum _{i}|({\hat{d}}_{i}^{(t+1)}-{\hat{d}}_{i}^{(t)})-({d}_{i}^{(t+1)}-{d}_{i}^{(t)})|$$

Collectively, this temporal metric complements the frame-wise spatial evaluation by assessing the stability and coherence of predicted depth sequences. It provides a quantitative basis for evaluating models on the dynamic and deformable scenarios that are characteristic of robotic-assisted laparoscopic surgery.

#### Models

Numerous approaches have adapted and fine-tuned general-purpose depth estimation models to the surgical domain, with notable gains in in-distribution accuracy^7^ The objective of the present benchmark is distinct and complementary: we evaluate the publicly released, off-the-shelf foundation models from which many of these surgical-specific approaches are derived or have been used as baselines. Reporting their out-of-the-box performance on DRENDS^1^ establishes a common, reproducible reference point against which any domain-adapted method can be positioned. To this end, we benchmark nine publicly released depth estimation models, grouped into three classes that differ fundamentally in what their outputs represent and, consequently, in the alignment required to compare them against ground truth: relative monocular, metric monocular, and metric stereo.

#### Relative monocular depth

**Monodepth2**^22^, **MiDaS**^23^, and the relative variant of **Depth Anything Model V2**^24^ (**DAMv2**) are trained with a scale-and-shift-invariant (SSI) objective in inverse-depth space. The SSI loss, introduced for MiDaS^23^, computes a closed-form per-image scale and shift that best aligns the network output to the ground-truth inverse depth on the valid pixels, and subtracts that fit out before scoring the residual; the network is therefore explicitly trained to discard the scale and shift of its inverse-depth output. Monodepth2 is self-supervised on monocular video via photometric reprojection and is scale-ambiguous only, since the reprojection loss fixes the zero point of disparity; MiDaS and Depth Anything V2 are trained on heterogeneous mixtures of depth datasets and are ambiguous up to both scale and shift. The output of all three models is therefore affine-related to the ground-truth inverse depth, not to depth itself. It is important to notice that we accordingly adopt the inverse-depth alignment under which these models were originally trained: we solve Eq. 7 after replacing d and $\hat{d}$ with 1/d and $1/\hat{d}$, fit $({s}^{\ast },{t}^{\ast })$ in inverse-depth space, apply the linear map there, and invert the result back to depth before computing metrics. Because the raw output of these models is in arbitrary inverse-depth units, metric error (MAE, RMSE, AbsRel, ${\delta }_{k}$) is undefined before alignment and is reported only for the aligned variant.

#### Metric monocular depth

**ZoeDepth**^25^, **Depth Pro**^26^, and the metric variant of **Depth Anything V2**^24^ (DAMv2-metric) predict depth in absolute units (metres). ZoeDepth couples a MiDaS-style SSI backbone with metric bin heads that express depth as a weighted combination of learnable bin centres; the relative pretraining provides cross-domain generalisation while the bin heads, fine-tuned on labelled metric data, anchor the output to a physical scale and route between domains at inference time. Depth Pro is a multi-scale vision transformer trained with large-scale supervised metric depth on a curated dataset mixture, designed to produce sharp object boundaries and to remain metric without external intrinsics: a focal-length head is trained jointly and supplies the camera parameters at inference when none are provided. DAMv2-metric reuses the self-distilled relative DAMv2 backbone (trained on tens of millions of unlabelled images plus a curated labelled subset) and fine-tunes it for metric prediction on synthetic indoor RGB-D data. All three were trained outside the endoscopic domain, so a residual systematic bias on DRENDS^1^ is expected; we therefore report the raw predictions, which represent the deployable accuracy a user obtains by running the off-the-shelf model, and additionally an aligned variant using Eq. 7 in which a per-sequence linear fit absorbs that residual bias. The gap between raw and aligned metrics quantifies the domain shift between each model’s training distribution and DRENDS^1^.

#### Metric stereo depth

**RAFT-Stereo**^27^, **UniMatch**^28^, and **FoundationStereo**^29^ predict per-pixel horizontal disparity δ between the rectified stereo pair. RAFT-Stereo iteratively refines disparity on a multi-level correlation volume using a recurrent update operator. UniMatch treats stereo matching as a dense correspondence problem within a unified transformer architecture shared with optical flow and depth estimation, using cross-attention between left and right feature maps to compute disparity in a single forward pass. FoundationStereo is a transformer-based zero-shot stereo model that builds on a vision foundation backbone and is trained on a large mixture of synthetic and real stereo data, generalising without per-domain fine-tuning. Disparity is converted to metric depth using the standard pinhole relation:

9 $$\begin{array}{lll}{\hat{d}}_{i}=\frac{f\cdot B}{{\delta }_{i}}, & f={P}_{{\rm{left}}}[0,0], & B=-\frac{{P}_{{\rm{right}}}[0,3]}{f}\cdot {10}^{3},\end{array}$$

Where *f* is the rectified focal length in pixels, and *B* is the rectified baseline in millimetres. Both are calculated per sequence from the rectified projection matrices ${P}_{{\rm{left}}},{P}_{{\rm{right}}}\in {{\mathbb{R}}}^{3\times 4}$ that are provided in the calibration files. The use of rectified projection matrices (rather than the raw intrinsics *K*) is essential: the rectified focal length differs from the unrectified focal length by a homography-dependent factor, and interchanging them introduces a silent bias to every predicted depth by a multiplicative constant. As with the metric monocular models, we report both the raw stereo predictions and a per-sequence depth-space aligned variant to make any residual calibration bias explicit.

#### Benchmark results

**Fig. 8:**
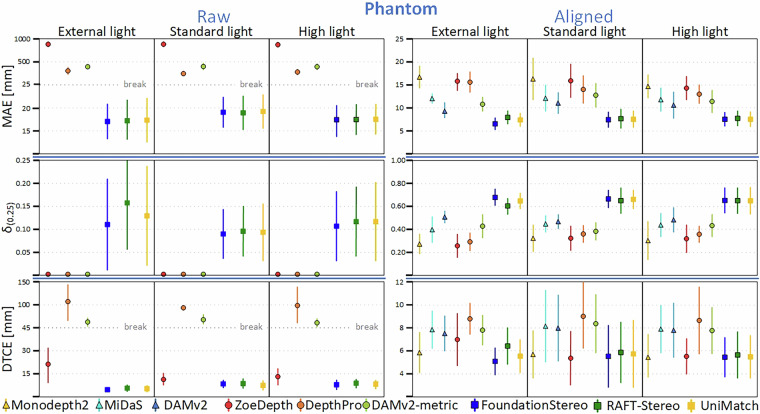
Benchmarking over DRENDS-Phantom under all illumination (*External Light*, *Standard Light*, and *High Light*). Results from all different sequences are combined and reported per model, illumination and output type (raw and aligned).

**Fig. 9:**
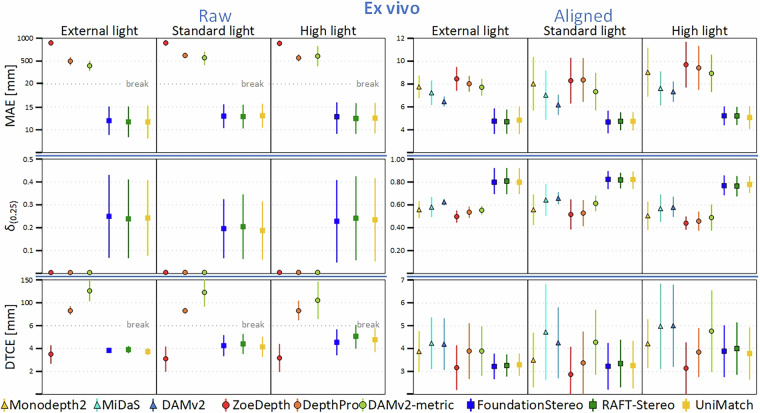
Benchmarking over DRENDS-*Exvivo* under all illumination (*External Light*, *Standard Light*, and *High Light*). Results from all different sequences are combined and reported per model, illumination and output type (raw and aligned).

**Fig. 10:**
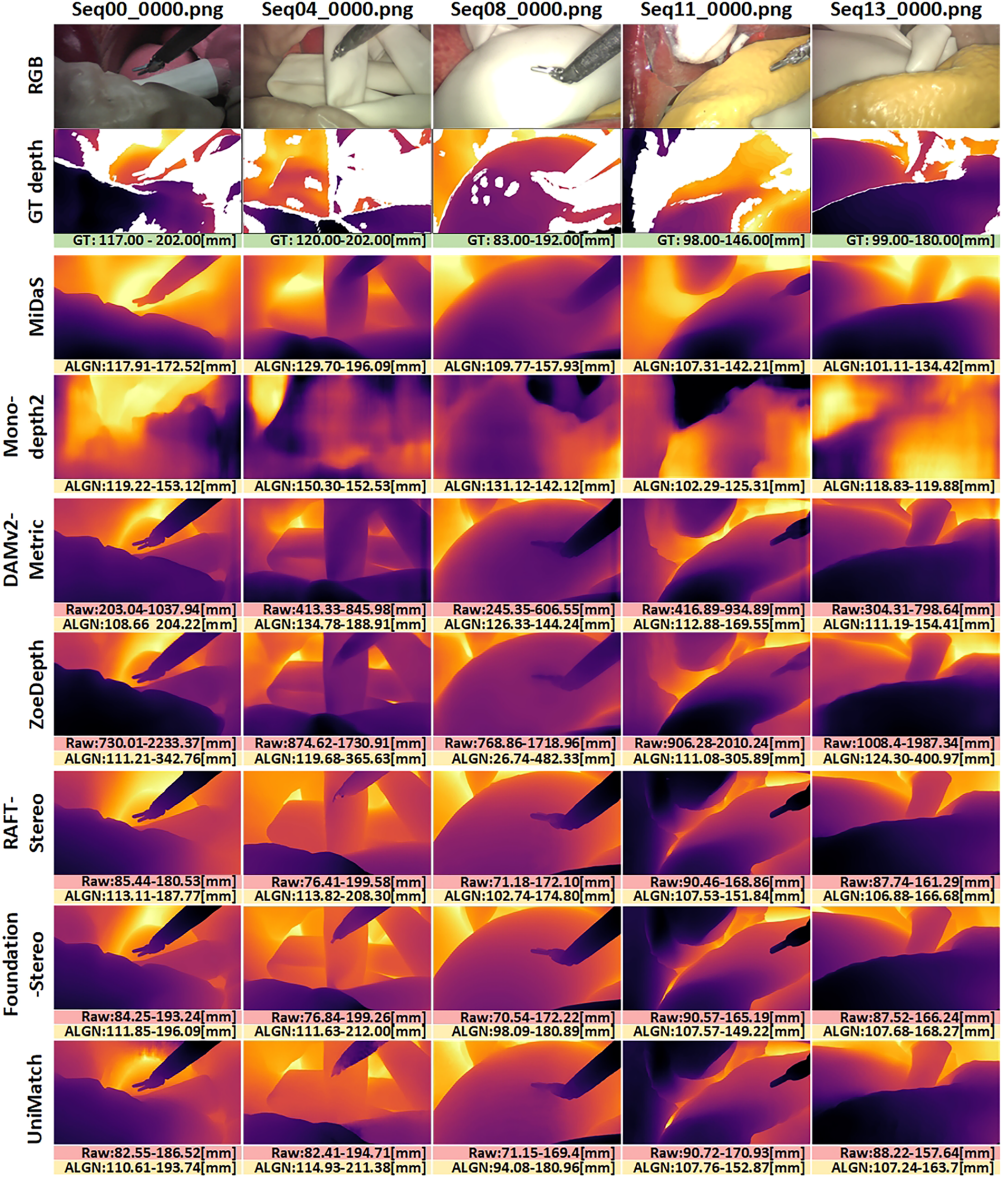
Qualitative depth estimation comparison of SOTA models over an image frame per organ in the DRENDS panthom subset. The depth range for ground truth (GT), unaligned (RAW), and aligned (ALGN) is presented for each frame.

**Fig. 11:**
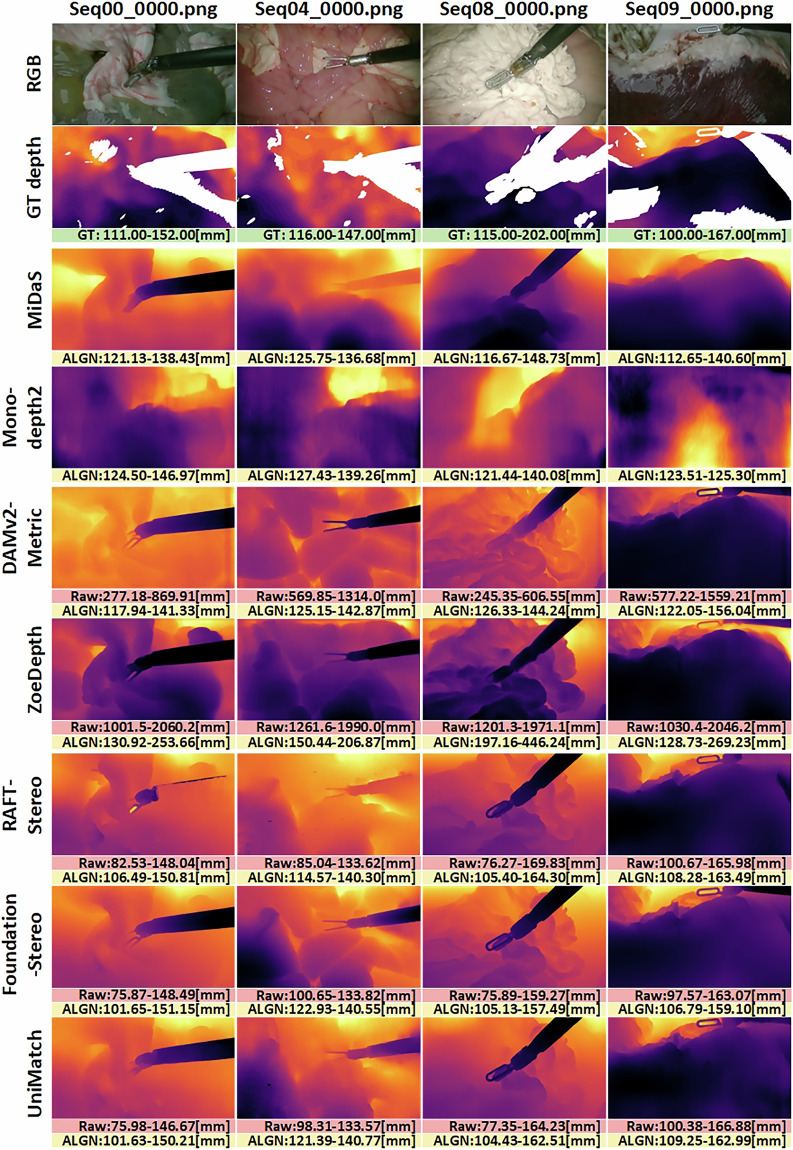
Qualitative depth estimation comparison of SOTA models over an image frame per organ in the DRENDS *ex vivo* subset. The depth range for ground truth (GT), unaligned (RAW), and aligned (ALGN) is presented for each frame.

We evaluate the nine models on both DRENDS^1^ subsets for raw and aligned predictions, and report spatial accuracy (MAE, RMSE, RMSE-log, AbsRel), threshold accuracy (δ), and temporal consistency (DTCE), with each metric computed across the three illumination conditions. Tables 3 and 4 report the Phantom and *ex vivo* results, Figs. 8 and 9 present the corresponding per-illumination breakdown, and Figs. 10 and 11 present qualitative results of the models of one image per anatomy.

**Table 3:** Comparison of depth estimation SOTA methods across various metrics on DRENDS-Phantom sequences.

Model	MAE (↓) (in *mm*)	RMSE (↓) (in *mm*)	RMSE-log (↓)	AbsRel (↓)	Depth Accuracy (↑)			DTCE (↓) (in *mm*)
					*δ*^0.25^	*δ*^0.50^	*δ*^0.75^	
**Non aligned (RAW)**								
ZoeDepth	849.39 ± 25.60	849.98 ± 25.01	2.00 ± 0.11	6.47 ± 0.86	0.00 ± 0.00	0.00 ± 0.00	0.00 ± 0.00	13.34 ± 10.21
DepthPro	276.62 ± 67.66	286.23 ± 67.17	1.06 ± 0.17	2.05 ± 0.54	0.00 ± 0.01	0.01 ± 0.01	0.01 ± 0.02	102.20 ± 30.18
DAMv2-Metric	378.38 ± 52.12	388.44 ± 53.25	1.32 ± 0.12	2.81 ± 0.48	0.00 ± 0.00	0.00 ± 0.00	0.00 ± 0.00	55.82 ± 9.27
FoundationStereo	17.86 ± 4.33	21.09 ± 4.61	0.16 ± 0.03	0.13 ± 0.02	0.10 ± 0.09	0.41 ± 0.14	0.77 ± 0.10	6.02 ± 2.01
RAFT-Stereo	17.98 ± 4.53	21.35 ± 4.78	0.17 ± 0.03	0.13 ± 0.02	0.12 ± 0.08	0.41 ± 0.15	0.75 ± 0.12	6.83 ± 2.27
UniMatch	18.00 ± 4.66	21.23 ± 4.81	0.16 ± 0.03	0.13 ± 0.02	0.11 ± 0.09	0.41 ± 0.15	0.76 ± 0.12	6.03 ± 1.99
**Aligned (ALGN)**								
Monodepth2	15.79 ± 3.18	19.94 ± 3.57	0.14 ± 0.02	0.11 ± 0.02	0.30 ± 0.11	0.57 ± 0.11	0.78 ± 0.09	5.59 ± 1.87
MiDaS	12.02 ± 2.12	15.62 ± 2.75	0.11 ± 0.02	0.09 ± 0.02	0.42 ± 0.09	0.70 ± 0.09	0.88 ± 0.05	7.97 ± 2.44
DAMv2	10.48 ± 2.17	13.73 ± 2.83	0.10 ± 0.02	0.08 ± 0.01	0.48 ± 0.07	0.77 ± 0.07	0.91 ± 0.04	7.72 ± 2.36
ZoeDepth	15.34 ± 2.85	19.09 ± 3.18	0.14 ± 0.02	0.11 ± 0.02	0.30 ± 0.11	0.56 ± 0.13	0.80 ± 0.08	5.93 ± 2.21
DepthPro	14.27 ± 2.58	17.75 ± 2.80	0.13 ± 0.02	0.11 ± 0.02	0.33 ± 0.08	0.61 ± 0.11	0.82 ± 0.07	8.87 ± 2.46
DAMv2-Metric	11.75 ± 2.34	14.94 ± 2.79	0.11 ± 0.02	0.09 ± 0.02	0.41 ± 0.08	0.71 ± 0.09	0.89 ± 0.06	8.00 ± 1.99
FoundationStereo	**7.24** ± **1.43**	**10.76** ± **2.19**	**0.08** ± **0.01**	**0.05** ± **0.01**	**0.68** ± **0.08**	**0.91** ± **0.03**	**0.95** ± **0.02**	**5.32** ± **1.96**
RAFT-Stereo	7.82 ± 1.53	11.43 ± 2.07	**0.08** ± **0.01**	0.06 ± 0.01	0.64 ± 0.09	0.88 ± 0.04	0.94 ± 0.02	6.00 ± 2.16
UniMatch	7.43 ± 1.48	10.91 ± 2.10	**0.08** ± **0.01**	**0.05** ± **0.01**	0.66 ± 0.08	0.90 ± 0.04	**0.95** ± **0.02**	5.48 ± 2.03

**Table 4:** Comparison of depth estimation SOTA methods across various metrics on DRENDS-*Ex vivo* sequences.

Model	MAE (↓) (in *mm*)	RMSE (↓) (in *mm*)	RMSE-log (↓)	AbsRel (↓)	Depth Accuracy (↑)			DTCE (↓) (in *mm*)
					*δ*^0.25^	*δ*^0.50^	*δ*^0.75^	
**Non aligned (RAW)**								
ZoeDepth	870.28 ± 3.76	870.35 ± 3.77	2.05 ± 0.03	6.79 ± 0.25	0.00 ± 0.00	0.00 ± 0.00	0.00 ± 0.00	**3.32** ± **1.16**
DepthPro	572.88 ± 174.42	575.93 ± 173.78	1.63 ± 0.27	4.46 ± 1.41	0.00 ± 0.00	0.00 ± 0.00	0.00 ± 0.00	121.72 ± 38.07
DAMv2-Metric	597.51 ± 80.97	602.39 ± 81.98	1.71 ± 0.13	4.64 ± 0.68	0.00 ± 0.00	0.00 ± 0.00	0.00 ± 0.00	84.58 ± 11.62
FoundationStereo	12.44 ± 3.00	14.18 ± 2.80	0.12 ± 0.03	0.10 ± 0.02	0.22 ± 0.17	0.63 ± 0.19	0.92 ± 0.04	4.37 ± 1.15
RAFT-Stereo	12.46 ± 3.00	14.17 ± 2.79	0.12 ± 0.03	0.10 ± 0.02	0.22 ± 0.17	0.62 ± 0.19	0.92 ± 0.04	4.50 ± 1.16
UniMatch	12.51 ± 2.94	14.18 ± 2.66	0.12 ± 0.02	0.10 ± 0.02	0.22 ± 0.16	0.62 ± 0.19	0.92 ± 0.04	4.22 ± 1.10
**Aligned (ALGN)**								
Monodepth2	8.28 ± 2.00	10.43 ± 2.56	0.08 ± 0.02	0.06 ± 0.02	0.53 ± 0.11	0.85 ± 0.09	0.96 ± 0.04	3.88 ± 1.09
MiDaS	7.30 ± 1.56	9.11 ± 1.90	0.07 ± 0.02	0.06 ± 0.01	0.59 ± 0.11	0.88 ± 0.07	0.98 ± 0.02	4.66 ± 1.79
DAMv2	6.75 ± 0.92	8.47 ± 1.20	0.07 ± 0.01	0.05 ± 0.01	0.62 ± 0.07	0.91 ± 0.05	0.98 ± 0.02	4.51 ± 1.59
ZoeDepth	8.76 ± 1.81	10.85 ± 2.31	0.08 ± 0.02	0.07 ± 0.02	0.49 ± 0.09	0.84 ± 0.08	0.95 ± 0.04	3.07 ± 1.16
DepthPro	8.57 ± 1.76	10.65 ± 2.28	0.08 ± 0.02	0.07 ± 0.02	0.50 ± 0.09	0.84 ± 0.08	0.96 ± 0.04	3.67 ± 1.26
DAMv2-Metric	7.92 ± 1.53	9.89 ± 1.95	0.08 ± 0.02	0.06 ± 0.01	0.55 ± 0.09	0.86 ± 0.07	0.96 ± 0.03	4.35 ± 1.49
FoundationStereo	4.86 ± 0.96	6.56 ± 1.26	**0.05** ± **0.01**	**0.04** ± **0.01**	0.80 ± 0.09	**0.96** ± **0.03**	**0.98** ± **0.01**	3.44 ± 1.02
RAFT-Stereo	4.85 ± 0.95	6.56 ± 1.24	**0.05** ± **0.01**	**0.04** ± **0.01**	0.80 ± 0.08	**0.96** ± **0.03**	**0.98** ± **0.01**	3.53 ± 0.98
UniMatch	**4.78** ± **0.99**	**6.47** ± **1.28**	**0.05** ± **0.01**	**0.04** ± **0.01**	**0.81** ± **0.09**	**0.96** ± **0.03**	**0.98** ± **0.01**	3.42 ± 1.02

The metrics for the raw predictions cleanly separate the models by the source of their absolute scale. Stereo models are the closest to out-of-the-box relevance, achieving approximately 18 mm MAE on Phantom (Table 3) and 12 mm on *ex vivo* (Table 4) (AbsRel 0.10 and 0.13, respectively). On the other hand, the metric monocular models infer absolute scale from priors tied to their training distribution, which is far out of distribution for laparoscopic imagery. That learned scale produces errors of hundreds of millimetres (spatial and threshold columns in Tables 3 and 4), up to ~850 mm, with AbsRel between 2 and 7, and essentially no pixels within the tolerance bands ($\delta \approx 0$). The relative monocular models carry no absolute scale by construction and are undefined in the metric space; they are reported only after alignment.

Per-sequence alignment brings the prediction depth range into the GT range (Figs. 10 and 11) and exposes the geometric quality of each prediction. All models converge to low-millimetre error (Tables 3 and 4), confirming that the large raw gaps were a matter of shift and scale rather than structure. The stereo methods remain the most accurate, clustering near 7–8 mm MAE on Phantom and 5 mm on *ex vivo*, with the highest threshold accuracy. Among the monocular models, the relative variant of DAMv2 is the strongest (10.48 mm on Phantom, 6.75 mm on *ex vivo*), narrowly ahead of MiDaS and of its own metric counterpart. In general, off-the-shelf models recover scene geometry competently but in an out-of-distribution depth range.

Temporal consistency matters independently of absolute accuracy in surgical video, where frame-to-frame jitter undermines tracking, registration, and 3D reconstruction. The large raw DTCE of DepthPro and DAMv2-metric (Tables 3 and 4) is a combination of jitter and scale error; inter-frame motion is amplified, and it decreases once alignment restores the scale. Among the stable models, stereo methods stand out with the lowest DTCE under the raw protocol and remain among the most stable after alignment. ZoeDepth is an interesting case: despite having the worst spatial accuracy, its raw DTCE is among the lowest (13.3 mm on Phantom, 3.3 mm on *ex vivo*), because its scale error stays nearly constant across frames, showing that temporal smoothness and absolute accuracy are distinct properties.

The per-illumination breakdown shows that lighting is not a primary source of error. Within each family, the metrics vary only modestly across the external, standard, and high-illumination conditions (Figs. 8 and 9), and this variation is small relative to the differences between models and between subsets. The dominant limitations are therefore the domain shift and, for the monocular models, the absolute scale, rather than the photometric conditions under which the sequences were captured. Figures 10 and 11 present qualitative depth maps on representative phantom and *ex vivo* frames. Most methods recover the scene’s geometry at the wrong scale, as evidenced by their low alignment errors and depth ranges, but differ in their finer structure. The discrepancies concentrate in the regions with low-textured, homogeneous surfaces and strong specular highlights, where photometric cues are weak.

#### Benchmark conclusion

Our benchmark establishes a reproducible reference point for off-the-shelf depth estimation in robotic endoscopy and yields a clear practical verdict: stereo methods are the closest to being deployable out of the box, recovering depth via triangulation from the known rectified camera parameters; they provide a geometric reference for the surgical scenario without any learned scale. It is important to note that stereo methods still exhibit residual error, reflecting the difficulty of stereo correspondence in the surgical setting, where low-textured, homogeneous tissue and strong specular highlights diminish the photometric cues on which matching depends. Monocular models cannot produce metric depth out of the box, despite recovering the 3D shape of the scenario. Their gap then is not only the photometric challenges but a missing metric anchor, which suggests that adapting monocular models to surgery should focus on recovering scale through sparse metric cues, known camera geometry, or a lightweight calibration step rather than on re-learning the geometry they already capture.

Temporal consistency emerges as an axis of performance distinct from spatial accuracy, and any benchmark for surgical video should report it alongside spatial accuracy. ZoeDepth illustrates the decoupling most clearly: it is the least accurate model in absolute terms yet among the most temporally stable, because its scale error stays nearly constant across frames and therefore introduces little frame-to-frame jitter. A model can thus be poorly scaled yet smooth in time, or accurate yet unstable, so neither property can be inferred from the other. Here, temporal stability refers to a smooth and proportional response to camera movements and deformations (rigid and non-rigid). In downstream tasks such as tracking, registration, and 3D reconstruction, where stability matters as much as per-frame accuracy, both must be measured explicitly.

Finally, the two subsets isolate distinct facets of the problem. Although Phantom is synthetic, it is consistently the harder regime: across all models, the aligned errors are higher, the threshold accuracy is lower, and the temporal consistency is worse than on *ex vivo*. This follows from its wider depth range and its smooth, low-texture, specular surfaces, which magnify scale-sensitive errors and destabilise temporal predictions, whereas the *ex vivo* sequences, despite the greater visual complexity of real tissue and non-rigid deformation, span a narrower depth range and are correspondingly easier once scale is corrected. The dominant difficulty in this domain is therefore not visual realism but the geometric and photometric constraints imposed by endoscopy: a wide depth range (0 to 30 mm during a complete procedure), weak texture, and strong specular reflections. The off-the-shelf evaluation reported here is deliberately a baseline; the natural next step is to use DRENDS^1^ to develop and assess surgical-specific models that combine the relative backbones already capable of recovering geometry with the scale-recovery strategies this benchmark identifies as the missing ingredient.

## Discussion

This work introduced DRENDS^1^, a high-resolution stereo-endoscopic dataset with per-frame metric depth derived from a synchronised time-of-flight sensor, explicitly capturing tissue manipulation and non-rigid deformations. By providing calibrated multi-modal recordings, precise timestamps, and an open processing pipeline for rectification, occlusion handling, and depth-map generation, DRENDS^1^ addresses a key limitation of existing resources: the scarcity of depth ground truth in dynamic surgical scenarios. At the same time, the dataset reflects a set of deliberate design choices. Depth annotations inherit the physical constraints of active time-of-flight sensing, including increased noise at depth discontinuities, sensitivity to surface reflectance, and partial occlusions during tool-tissue interaction. The measurements are bounded by the manufacturer-specified accuracy of the sensor and are further stabilised during preprocessing through temporal and spatial filtering. Rather than attempting to compensate for these effects using an explicit parametric noise model, the processing pipeline applies lightweight filtering designed to suppress measurement noise while preserving the natural characteristics of the sensor observations. Furthermore, the data were acquired from a single experimental setup involving phantom and *ex vivo* tissue, which limits the diversity of anatomical structures across specimens. Rather than aiming to exhaustively represent the full diversity of surgical procedures and patients, DRENDS^1^ focuses on controlled scenarios that emphasise clinically relevant challenges for depth estimation such as specular surfaces, deformable tissue, and dynamic interactions. While these scenarios do not fully reproduce the variability of *in vivo* surgical environments, they enable systematic and reproducible analysis of metric accuracy and temporal consistency under conditions that are difficult to isolate in unconstrained clinical settings, positioning DRENDS^1^ as a principled step toward bridging the gap between synthetic benchmarks and *in vivo* deployment.

We anticipate that DRENDS^1^ will accelerate research in (i) metric depth estimation with reliable scale, (ii) temporally coherent depth prediction under deformation, and (iii) hybrid approaches that combine learning with surgical priors and multi-sensor constraints. By making the dataset and processing tools publicly available, we aim to support reproducible comparisons and encourage the development of methods that can translate into dependable 3D perception for robotic-assisted surgery.

## Usage Notes

The DRENDS^1^ dataset is organised into per-sequence folders containing stereo videos, time-of-flight point clouds, and calibration metadata. Users can extract the archives with any standard decompression tool and load the modalities using the companion Python utilities provided in the project repository. The codebase includes functions for reading point clouds and frames, applying the provided calibration parameters, and obtaining rectified frames, depth maps and binary masks. Detailed usage examples and scripts are available on the project’s webpage (https://l0za007.github.io/DRENDS).

## Data Availability

The DRENDS^1^ dataset is publicly available on Zenodo under the project identifier Zenodo-17598453. The data are released under a CC BY-4.0 licence to permit reuse and adaptation with attribution.

## Code availability

The processing pipeline, including calibration modules and depth-map generation scripts, is openly available in the accompanying GitHub repository(https://github.com/L0za007/DRENDS). The benchmarking code is also openly available in the complementary GitHub repository (https://github.com/L0za007/DRENDS_Eval). The repositories also include the exact scripts used to generate the depth maps and the evaluation results reported in the paper, ensuring full reproducibility. The code is released under the Apache 2.0 licence.
